# Mutations of the *Drosophila* Myosin Regulatory Light Chain Affect Courtship Song and Reduce Reproductive Success

**DOI:** 10.1371/journal.pone.0090077

**Published:** 2014-02-26

**Authors:** Samya Chakravorty, Hien Vu, Veronica Foelber, Jim O. Vigoreaux

**Affiliations:** 1 Department of Biology, The University of Vermont, Burlington, Vermont, United States of America; 2 Biochemistry Undergraduate Program, The University of Vermont, Burlington, Vermont, United States of America; 3 Department of Molecular Physiology and Biophysics, The University of Vermont, Burlington, Vermont, United States of America; Universitaet Regensburg, Germany

## Abstract

The *Drosophila* indirect flight muscles (IFM) rely on an enhanced stretch-activation response to generate high power output for flight. The IFM is neurally activated during the male courtship song, but its role, if any, in generating the small amplitude wing vibrations that produce the song is not known. Here, we examined the courtship song properties and mating behavior of three mutant strains of the myosin regulatory light chain (DMLC2) that are known to affect IFM contractile properties and impair flight: (i) D*mlc*2^Δ2–46^ (Ext), an N-terminal extension truncation; (ii) D*mlc*2^S66A,S67A^ (Phos), a disruption of two MLC kinase phosphorylation sites; and (iii) D*mlc*2^Δ2–46;S66A,S67A^ (Dual), expressing both mutations. Our results show that the *Dmlc2* gene is pleiotropic and that mutations that have a profound effect on flight mechanics (Phos and Dual) have minimal effects on courtship song. None of the mutations affect interpulse interval (IPI), a determinant of species-specific song, and intrapulse frequency (IPF) compared to Control (D*mlc*2*^+^* rescued null strain). However, abnormalities in the sine song (increased frequency) and the pulse song (increased cycles per pulse and pulse length) evident in Ext males are not apparent in Dual males suggesting that Ext and Phos interact differently in song and flight mechanics, given their known additive effect on the latter. All three mutant males produce a less vigorous pulse song and exhibit impaired mating behavior compared to Control males. As a result, females are less receptive to Ext, Phos, and Dual males when a Control male is present. These results open the possibility that DMLC2, and perhaps contractile protein genes in general, are partly under sexual selection. That mutations in DMLC2 manifest differently in song and flight suggest that this protein fulfills different roles in song and flight and that stretch activation plays a smaller role in song production than in flight.

## Introduction

Muscle is well known for its ability to generate force through a contractile mechanism that involves sliding of myosin-containing thick filaments pass actin-containing thin filaments. A fundamental outcome of the force is movement, principal among which is animal locomotion. The ability to fly, present in the majority of insect species including *Drosophila*, is generally considered one of the main driving forces in the evolution of insects due to the fact that it facilitates escape from predators, dispersal, colonization of new niche, and subsequent speciation [1], [2]. Another factor that contributes to speciation is acoustic communication and the role it plays in pre-mating reproductive isolation in many animals [3]–[7], including most insects [8], [9]. In *Drosophila*, males generate species-specific courtship songs of rhythmic pulses and sinusoidal hums generated by small amplitude wing vibrations [10]–[14]. During singing, the thoracic flight musculature, consisting of direct flight muscles and indirect flight muscles (IFM), is neurally activated [15], [16]. However the precise roles of these muscles in generating the song have not been investigated. There is strong evidence the IFM has evolved mechanisms that enable exceptionally high wing-beat frequencies (approximately 200 Hz in *Drosophila*) required for powered flight [17]. In contrast, *Drosophila* mating does not occur aerially and therefore the males do not need to overcome drag and lift forces during singing. Furthermore, males beat only one wing at a time during singing (reviewed in [12]) with an amplitude that is 1/4^th^ of that during normal flight [18]. Courtship song carrier frequencies reportedly have broad distributions with the *D. melanogaster* intrapulse song frequency (IPF) ranging from 150–350 Hz [19], with a median value of 240 Hz [20] and the sine song frequency (SSF) ranging from 110–185 Hz [21], with a median value of 130 Hz [22]. In comparison, the wing beat frequency during flight varies from 190–230 Hz [23]. It is not known how the different wing beat frequency ranges are generated by the same musculature. Therefore, IFM provides an excellent system to understand the contractile mechanisms of behavioral outputs with unique power output requirements, especially with song being very distinct from flight from an ecologocial, evolutionary, and physiological standpoint.

In order to understand how muscle protein genes are utilized for the two distinct IFM-driven behaviors, and to gain insight into the contractile mechanism used for song generation, we tested the effect of mutations of the *Drosophila* myosin regulatory light chain (DMLC2) (see Figure 1 in [24]), a highly conserved thick filament protein [25]. Two mutations, a truncation of the 46 amino acid N-terminal extension (D*mlc*2^Δ2–46^ or Ext) and alanine substitutions of two myosin light chain kinase phosphorylation sites (D*mlc*2^S66A,S67A^ or Phos) have no major effect on IFM ultrastructure [24], [26]–[30], thus enabling an understanding of how DMLC2 influences courtship song mechanics without the confounding effect that structural abnormalities may contribute. The mutations are known to have a large effect on myosin kinetics, wing beat frequency, and flight performance [24],without having any major effect on calcium activated isometric tension [26]–[28]. D*mlc*2^Δ2–46^ (Ext) reduces flight ability, wing beat frequency, and the frequency of maximum power output by moving the myosin heads farther away from actin target zones [24]. D*mlc*2^S66A,S67A^ (Phos) further reduces all three aforementioned parameters by increasing the angular mobility of myosin heads which effectively reduces the proportion of myosin heads that are properly oriented towards actin target zones for strong binding [24], [30]. The effects of the individual mutations are further compounded when expressed together, as is the case in the dual mutant *Drosophila* transgenic line D*mlc*2^Δ2–46;S66A,S67A^ (Dual) [24], [30]. This mutant shows alterations in both myosin head orientation to the thin filament, and alignment to target zones. In addition, Miller et al [24] reported that Dual flies are unable to fly or beat their wings during the tethered flight assay.

**Figure 1:**
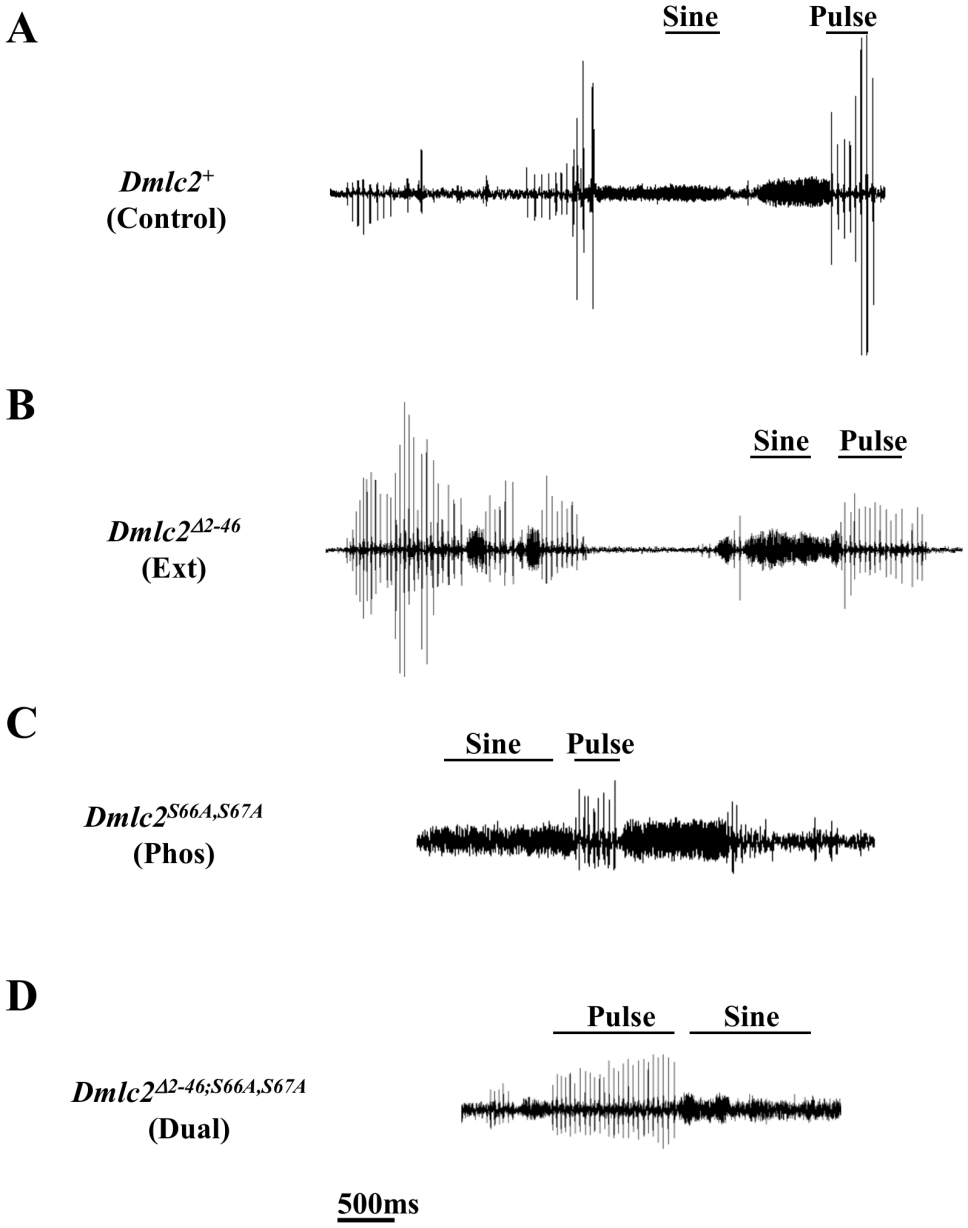
Courtship song oscillogram samples of control and mutant lines. Courtship song samples from transgenic males (A) Control, (B) Ext (C) Phos, and (D) Dual. In all cases, male courtship song was induced by the presence of a wild type (Oregon R) virgin female *D. melanogaster*. All strains produce sine song and pulse song. Song recording was done at 22°C and 70% humidity in a dark room, with the only light source inside the song recording chamber [59], [60]. The samples here were retrieved with Audacity software.

The above findings on the DMLC2 mutations indicate that the N-terminal extension and the phosphorylation of serines 66 and 67 serve to enhance power output and improve flight performance. In this study, we examined the effect of Ext, Phos, and Dual on courtship song properties, mating behavior, and reproductive success. We report that males from all three DMLC2 mutant strains are capable of generating a courtship song with sine and pulse elements. Deletion of the N-terminal extension affects several song parameters; however, most song parameters are normal in Dual. The results indicate that the two attributes of DMLC2, N-terminal extension and phosphorylation, interact very differently in the mechanisms responsible for flight and song.

## Results

### Tethered *Dmlc2* Mutant Males Do Not Beat their Wings

Miller et al. [24] had shown that females of the single mutants (Ext, Phos) are flight impaired and have a lower wing beat frequency compared to Control females, while Dual mutant females are flightless and produce no wing beat (Table 1 and also Table 1 in [24]). Since this study focuses on the male courtship song, we first tested the mutant males’ wing beat frequency during tethered flight. No difference was found in comparing the tethered wing beat frequencies of the males with the females of the same mutant strain (Table 1). Also, there was no sex difference in flight abilities of the mutant males (data not shown).

**Table 1:** Summary of female and male tethered flight wing beat frequency.

Line	Female wing beat frequency (Hz)^1^	Male wing beat frequency (Hz)
*Dmlc2^+^*(Control)	202±3 (52)	196±2 (10)
*Dmlc2^Δ2–46^*(Ext)	165±2* (44)	170±3* (10)
*Dmlc2^S66A,S67A^*(Phos)	158±3* (11)	168±7* (10)
*Dmlc2^Δ2–46;S66A,S67A^* (Dual)	0±0* § (30)	0±0* § (10)

1 Values from ref [24] **.** All values are mean ± SEM. Numbers in parenthesis indicate number of flies tested. Temperature = 22°C.

*Significant difference from *Dmlc2^+^*.

§ Significant difference from *Dmlc2^Δ2–46^* and *Dmlc2^S66A,S67A^*.

### Flight Compromised *Dmlc2* Mutant Males are Capable of Generating Courtship Song

**Figure 2:**
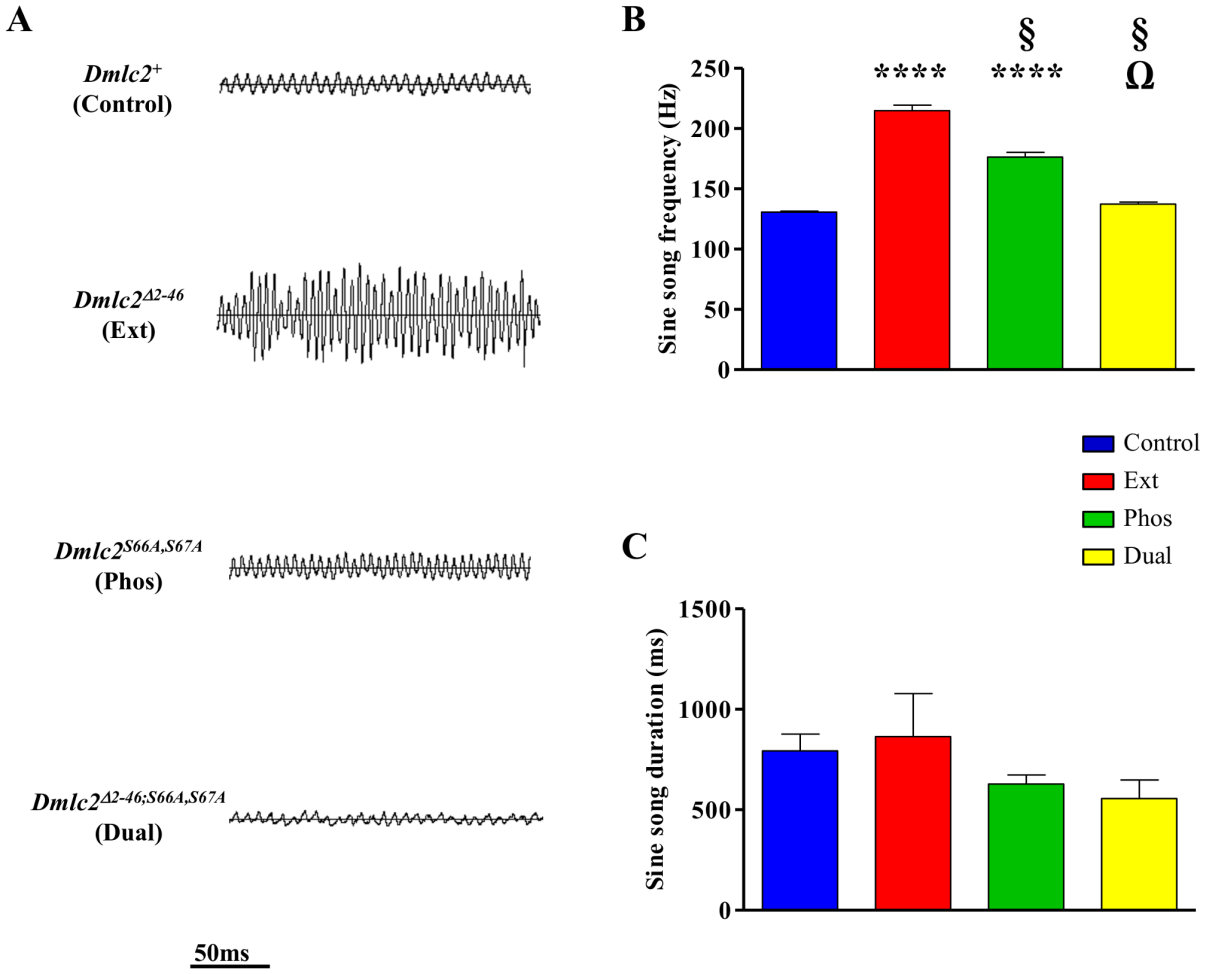
*Dmlc2* mutations affect sine song frequency. (A) Representative sine song oscillograms from Control, Ext, Phos and Dual males are shown (top to bottom panels, respectively). (B) Compared to Control (blue), sine song frequency is significantly higher in Ext (red) and Phos (green) mutants but similar in the Dual mutant (yellow). Also note that Ext mutant sings with a significantly higher sine song frequency compared to Phos and Dual mutants. (C) Sine song burst duration (SDUR) is similar for all the lines. See Materials and Methods and ref [76] for details and retrieval method of the sine song parameters. *n* = 7–8 males for each line. *(p<0.0001), § (p<0.0001) and Ω (p<0.0001) indicate significant difference from Control, Ext, and Phos respectively. Error bars indicate SEM.

**Figure 3:**
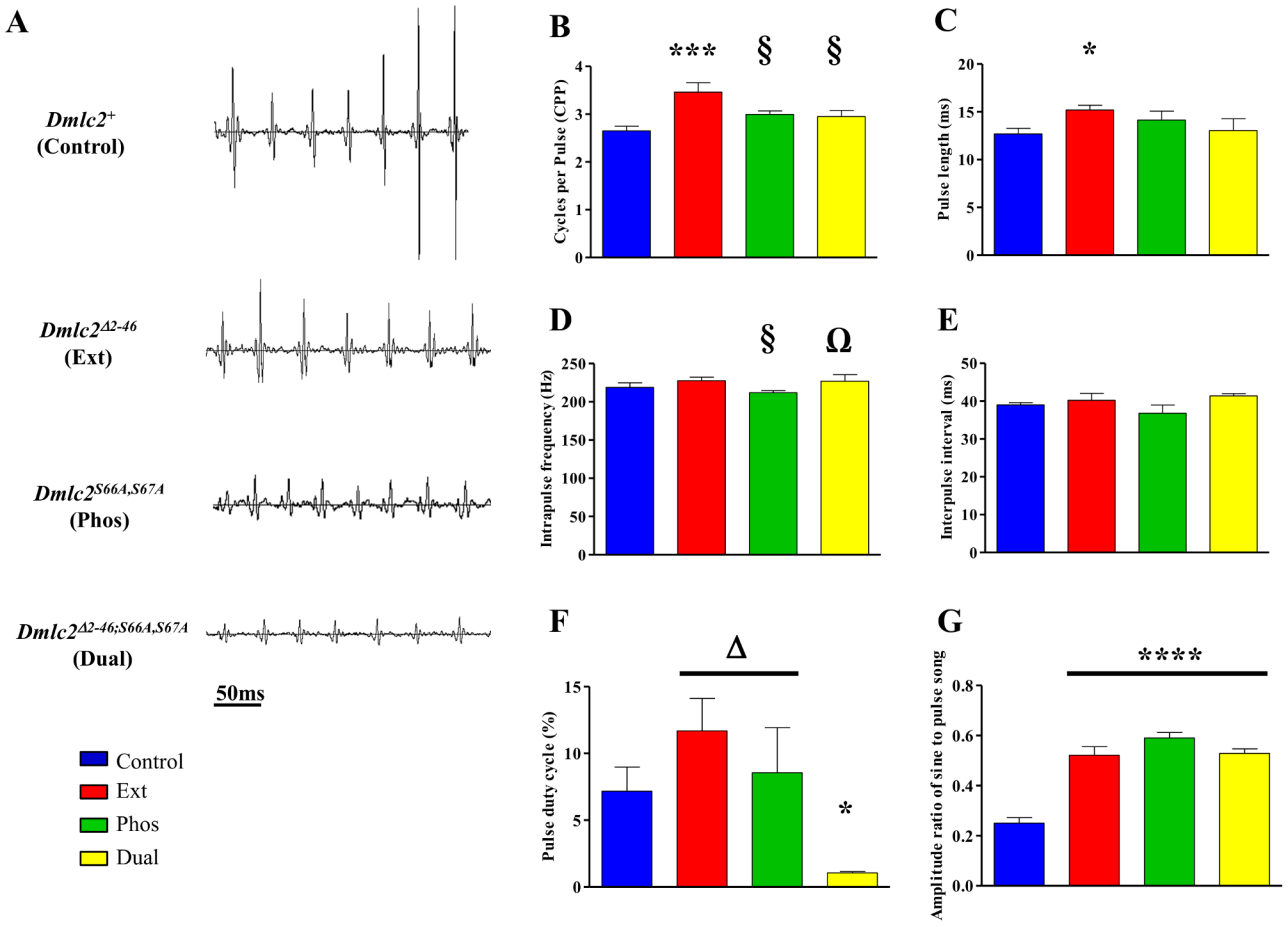
Effect of *Dmlc2* mutations on pulse song properties. (A) Representative pulse song oscillograms from Control, Ext, Phos and Dual males (top to bottom panels, respectively). (B) Phos and Dual males sing with similar cycles per pulse (CPP) and (C) pulse length (PL) compared to Control males. Ext males produce songs with higher CPP and longer PL. (D) All the mutant males produce pulse song with normal intrapulse frequency (IPF), with only the Phos mutants’ IPF showing a slight reduction compared to Ext and Dual. (E) None of the mutations affect interpulse interval. (F) The Dual mutant has significantly reduced pulse duty cycle compared to Control, Ext, and Phos. (G) Amplitude ratio (AMP-RT) of consecutive sine to pulse song is significantly higher in individual (Ext, Phos) and Dual mutants compared to Control. *n* = 7–8 males for each line. *(p<0.05), ***(p<0.001), ****(p<0.0001) indicate significant differences from Control. § (p<0.05), Ω (p<0.05) and Δ (p<0.05) indicate significant difference from Ext, Phos and Dual mutants, respectively. Error bars indicate SEM.

All the mutant males (Ext, Phos and Dual) are capable of generating pulse songs and sine songs as shown by representative oscillograms (Figures1, 2A, and 3A) and the song audio clips (Audios S1, S2, S3, and S4). The amplitudes of the sine and pulse songs are influenced by the relative position of the singing male to the microphone. Therefore, the differences in amplitude evident in the figures do not represent measurable differences in song quality. A more accurate parameter is the amplitude ratio of sine song to pulse song (AMP-RT; see Table S1 for definition of song parameters) during the transition between song types because both sine and pulse component of that transiton event is likely to occur at the same distance from the microphone [31].

### *Dmlc2* Single Mutations Increase Sine Song frequency

Males of the single mutant strains produce sine songs with abnormally high SSF (215±5 Hz for Ext and 176±4 Hz for Phos) compared to Control males (131±1 Hz). In contrast, the SSF of Dual males is similar to that of Control males (137±2 vs 131±1 Hz, respectively) indicating that the single mutations are mutually suppressing each other’s effects (Figure 2B). There was no significant difference in sine song burst duration (SDUR, Figure 2C) between the mutants and Control, suggesting that the single mutations do not affect the sine singing vigor.

### *Dmlc2* Mutations have Minor Effects on Pulse Song

The Ext mutant males produce more cycles per pulse (CPP, Figure 3B) and a concomitant increase in pulse length (PL, Figure 3C) compared to Control, Phos, and Dual. In contrast to the reduced or abolished flight wing beat frequencies in the *Dmlc2* mutant males (Table 1), the IPF (Figure 3D) is similar in all three mutants compared to Control. Phos males shows slightly lower IPF than Ext or Dual males, but not compared to Control males (Figure 3D). None of the *Dmlc2* mutations have an effect on inter-pulse interval (IPI, Figure 3E), one of the salient parameters under sexual selection in the *melanogaster* subgroup [14], [22], [32].

Pulse singing by Control males accounts for approximately 7.2% of the total courtship recording time or pulse duty ratio (PDC). While neither single mutant affects PDC, Dual males show approximately 85% decrease in PDC compared to Control males (Figure 3F). AMP-RT is higher for all three mutant males compared to Control males (Figure 3G) indicating either the mutants sing with a louder sine song or a softer pulse song.

### *Dmlc2* Mutations Affect Male Courtship Success

**Figure 4:**
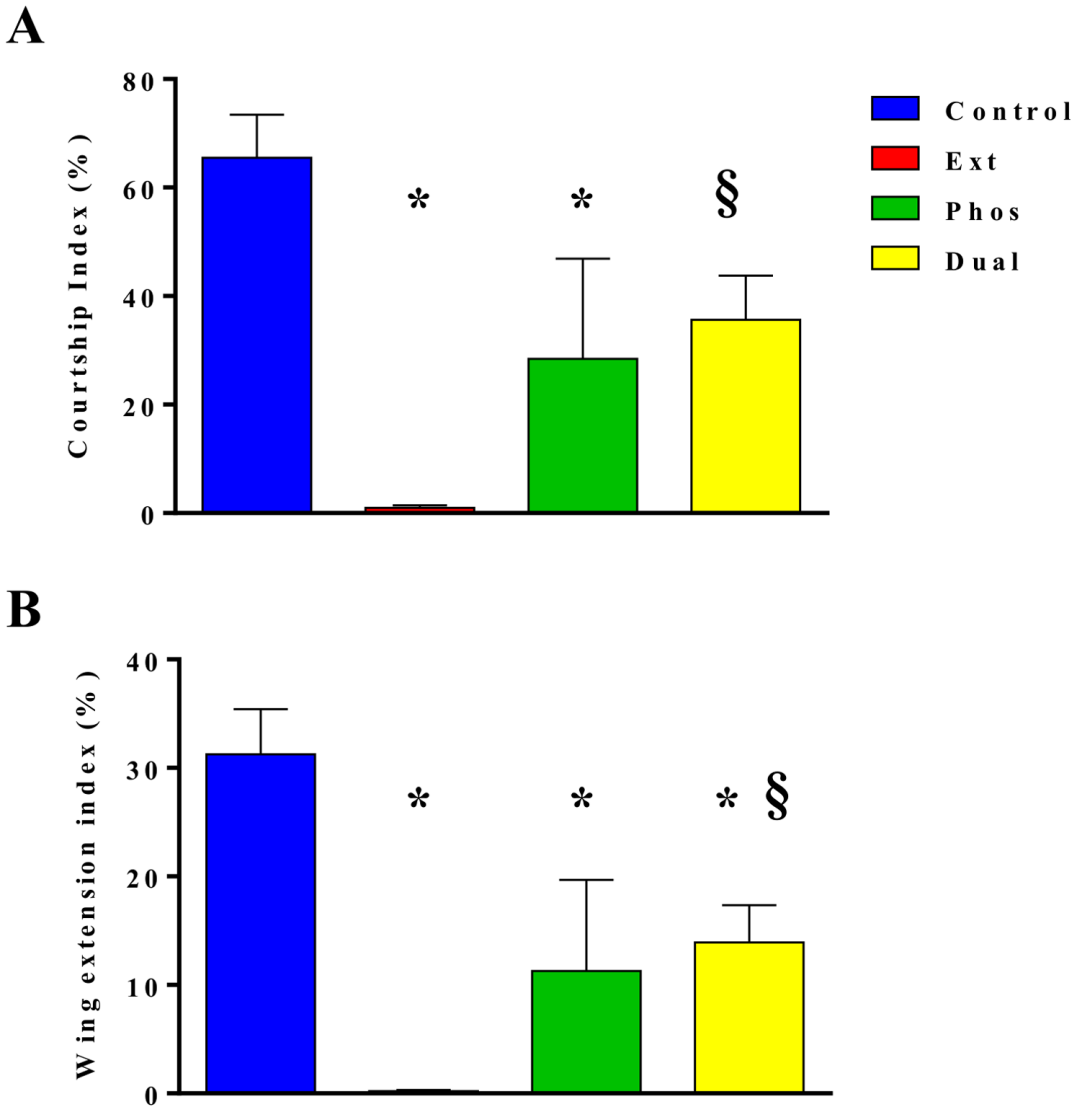
*Dmlc2* mutations affect courtship behavior. Single pair mating assays, consisting of a wild-type (OR) female and a male of Control or mutant strain were performed to assess male courtship vigor. (A) Courtship index (CI) and (B) Wing extension index (WEI). Ext and Phos mutants had significantly reduced CI, and Dual mutant’s CI is marginally reduced compared to Control (p = 0.054). Ext, Phos, and Dual mutants had significantly reduced WEI compared to Control (B). The Ext mutant, in particular, had the greatest reduction in CI and WEI compared to Control and Dual. *n* = 4–6 for each mating competition group. *(p<0.05) indicate significant difference from Control. § (p<0.05) indicate significant differences from Ext mutant. Error bars indicate SEM.

We tested males in single pair mating assays with wild type (OR) females and found that all the mutant males engage in courtship, albeit at much reduced levels compared to Control (Figure 4). The wing extension index (WEI) of all the mutants, especially Ext, is significantly reduced compared to Control, while the courtship index (CI) is reduced in Ext and Phos but not in Dual. Despite the handicaps in courtship performance, mutant males achieved 100% mating success within the courtship recorded time (see Materials & Methods).

**Figure 5:**
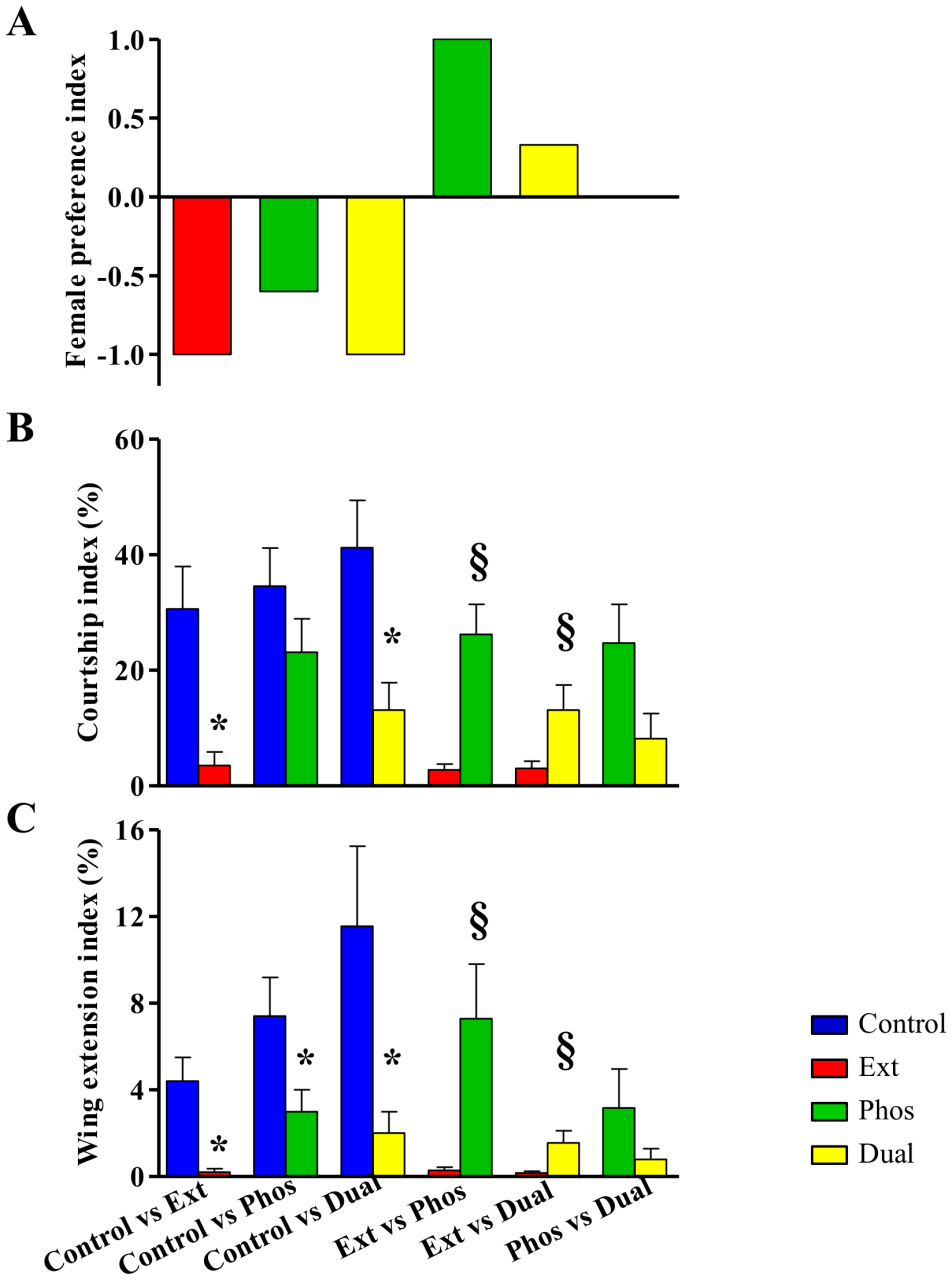
*Dmlc2* mutations affect male courtship vigor and female preference. (A) Female preference index (FPI) is the relative advantage of a male of specific genotype over a male of a different genotype, i.e., the excess number of copulations with a male of specific genotype divided by the total number of copulations [62]. Negative FPI indicate the Ext (red), Phos (green) and Dual (yellow) mutant males were out-competed by the Control male for female preference. Positive FPI indicate the Phos (green) and Dual (yellow) mutant males individually out-competed the Ext mutant males. There is no female preference for Phos or Dual males (FPI = 0). (B–C) Male courtship vigor in competitive mating situation was calculated via. courtship index (CI) and wing extension index (WEI). Ext and Dual mutants had significantly reduced CI and WEI but Phos mutant had only significantly reduced WEI compared to Control. In competition between mutants, Phos and Dual mutants have significantly higher CI and WEI compared to the Ext mutant. There is no difference between Phos and Dual. *n* = 20–30 for each mating competition group. *(p<0.05) indicate significant difference from Control. § (p<0.05) indicate significant differences from Ext mutant. No error bars in (A), (B–C) error bars indicate SEM.

To understand if the mutations’ effects on song and courtship affect their reproductive success, we conducted pairwise mating competitions of each mutant strain with a Control male for an OR wild-type female. All the mutant males were outcompeted by Control males as determined by female preference index (FPI) (Videos S1, S2, S3, Figure 5A). In the case of Ext and Dual, female choice was nearly 100% for Control male, whereas female preference was 60% higher for Control males than for Phos males. All mutant males exhibited lower courtship performance in the presence of a Control male, indicated by a lower CI and WEI (Ext and Dual) or lower WEI (Phos) (Figure 5B, C). Ext males are outcompeted by Phos and Dual males (Videos S4 and S5, Figure 5A) and show significantly reduced CI and WEI (Figure 5B, C). Dual males, which showed the least song aberrations compared to other mutants based on the number of parameters affected (Figure 3F,G), were able to outcompete Ext males (Video S5, Figure 5A) most likely due to their higher CI and WEI (Figure 5B, C). There is no female preference between Dual and Phos males when presented with the choice (Figure 5A). These mutant males exhibited similar CI and WEI when competing for an OR female (Figure 5B, C).

## Discussion

The *Dmlc2* mutations studied here have been extensively characterized for their effect on IFM and myofilament lattice structure, muscle fiber mechanical properties, and whole organismal flight performance [24], [26]–[30]. Here, we show that Ext, Phos, and Dual have distinct effects on courtship song and mating behavior, thus presenting an opportunity to elucidate the role of the IFM in non-flight behaviors. Importantly, this work contributes to our understanding of the extent to which IFM’s genetic and physiological pathways are shared between flight and courtship, two insect behaviors that are fundamental to survival and reproduction and that are likely shaped by separate evolutionary mechanisms. The inability of Ext and Phos mutant males to engage in significant courtship behavior and compete for female preference versus a non-mutant male raises the possibility that at least some regions of the *Dmlc2* gene are under sexual selection.

Our finding that all three DMLC2 mutant strains are capable of producing pulse and sine songs (Figure 1) show that these mutations have a less pronounced effect on courtship song generation compared to flight. This is most evident in the Dual mutant. A previous study showed that 100% of the Dual female flies tested were unable to fly when released into a flight chamber and none of the mutant female flies tested for tethered flight produced a wing beat [24]. A similar result was obtained here for Dual males (Table I). There are several explanations for the differences in functional effects. First, the power requirements for song are expected to be much less than for flight and therefore the mutations are less penetrant in the former than the latter. The high power output demands for flight are met by an enhanced stretch activation response. The enhancement is the result of biochemical and structural adaptations that bring about, among several features, a superfast myosin [33] and a beautifully ordered myofilament lattice structure [34], [35]. These features may play a smaller role in the mechanism for song generation since song generation carries neither the energetic nor aerodynamic demands of flight. Unlike flight, sine song production may not require activation of all motor units indicating that its power demands are lower than flight [15], [16], [36]. This is further supported by the observation that during courtship song, wing stroke amplitude (which correlates well with force [37], [38] and power output [39] of the flight system) is much lower than during flight [18]. Thus, it is possible that a subdued stretch activation is more than sufficient to generate the power for song production. Other observations that are consistent with this interpretation include the much slower rate of motorneuron firing during sine song as compared to flight, and the lower frequency of sine song as compared to flight [15].

Second, song production may rely largely or entirely on Ca^2+^ activation. This possibility is rendered more likely by the observation that neither Ext nor Phos affect calcium-activated tension in skinned IFM fibers [26]–[29].

Results showing that none of the DMLC2 mutations have an effect on IPI, the interval between successive pulses (Figure 3E) suggest that pulse initiation occurs normally. Ewing [15] has shown that muscle potentials during pulse song are more closely spaced than during sine song. Additionally, he showed that the activities of all the IFM motor units correlated with the timing of sound pulses and the muscle potentials functionally relate to the subsequent but not the preceding sound pulses. If, as expected, the muscle potentials lead to intracellular calcium release then it is reasonable to assume that pulses are regulated in a calcium-dependent manner. Further support for a calcium activated pulse comes from the observation that the timing between the muscle potential and the sound pulse (16 ms) is slightly longer than the timing between the flight starter jump and the first wing beat for flight (12 ms) [15], [40], a delayed response that may result from slow calcium diffusivity given the scarcity of sarcoplasmic reticulum in the IFM.

Recent studies have shown that *Drosophila* modulates IFM power output during flight through a calcium-dependent mechanism [36], [41], [42]. Work-loop analysis of IFM fibers showed that power output increases with increasing calcium concentration, indicating that a calcium-based mechanism can contribute to force above and beyond the force generated by stretch activation [41]. One explanation for this observation is that calcium is recruiting a different population of crossbridges than are being recruited through stretch, a distinction made possible by the co-expression of two isoforms of troponin C, one that responds to calcium (DmTnC1) and another that responds to stretch (DmTnC4) [43], [44]. The co-existence of two regulatory mechanisms may also explain why DMLC2 mutations affect flight mechanical parameters more drastically than song parameters.

The observed differences in pulse song among the mutant strains also point to a calcium-dependent mechanism. Unlike Phos and Dual, Ext shows longer PL compared to Control (Figure 3B, C). Calcium sensitivity of oscillatory work output is decreased in Ext IFM fibers [29]. Lower calcium sensitivity could result in reduced myosin head cooperativity and thin filament regulatory unit interactions, reducing the rate of force development and decay [45]–[48]. Thus, the longer pulses could be a reflection of deficiencies in the underlying activation kinetics. Another factor contributing to extended pulses in Ext could be the tendency of weakly bound cross-bridges to remain attached longer than normal cross-bridges [24]. However, it is not clear how long-lived weakly bound cross-bridges account for the other unique feature of Ext song, the increase in CPP. The N-terminal extension of the vertebrate essential light chain (ELC) has been shown to act as an internal load through its connection to the thin filament [49], [50]. If the RLC N-terminal extension fulfills a role similar to the ELC extension, as has been proposed [29], the reduction in internal load could affect myosin cycling kinetics and hence the number of cycles within a pulse per unit time.

The nature of the interaction between N-terminal mutation and the phosphorylation mutation in the Dual strain is intriguing. While the two mutations have been reported to combine additively to impair flight performance in Dual [24], no such effect is seen in any of the song properties. Quite the opposite, the high SSF evident in Ext and Phos is restored in Dual, and Phos suppresses the abnormal CPP and PL produced by Ext. It is possible that the observed flightlessness and absence of a wing beat is a behavioral problem and not a physiological problem (i.e., Dual flies are capable of beating their wings but unwilling to do so). We consider this possibility unlikely. As shown by Miller et al. [24], the maximum power output and the frequency of maximum power output are significantly reduced in active IFM fibers from Dual in comparison to active IFM fibers from Control, Ext, and Phos, when compressed to *in vivo* lattice spacing with Dextran T-500. As inertial power is the main contributor to mechanical power at flight initiation [23], the absence of a wing beat in Dual flies can be explained by the inability of their flight muscles to generate enough power, at the proper frequency, to overcome the inertia of the wings.

Despite the relatively mild effects on courtship song, the mutant males do not perform well in single pair mating assays with wild type OR females. Ext and Phos males, but not Dual males, showed significant reductions in CI while all three strains exhibited greatly reduced WEI compared to Control males (Figure 4). As the DMLC2 mutations are expressed in all muscles [26], [27], [30], [51], the behavioral differences are likely due to the effect of the mutations on direct flight muscles which may impair the male’s ability to extend out the wing (e.g., the second basalar responsible for moving the wing forward, [40]). In fact, most of the reduction in CI can be explained by the decrease in WEI as the two indices closely track each other.

In the courtship competition assays, wild-type females overwhelmingly preferred Control males over males of any of the three mutant strains (Figure 5). Ext and Dual males were rejected in 100% of the competitions while Phos males were rejected 60% more often than Control males when paired in competition. The low mating success of mutant males is likely due to the mutations’ effects on AMP-RT and WEI, the only two parameters that are affected in all the mutant strains. The reduction in AMP-RT can result from either an increase in the amplitude of the sine song or a decrease in the amplitude of the pulse song. Several studies have concluded that con-specific female choice is largely influenced by the quality of the pulse song, with the sine song playing little to no effect [22], [52], [53]. In the case of the competitions, a common observation was that Control males were more mobile and tended to gain close access to the female more quickly than mutant males (Videos S1–S3). Since males rely on a loud pulse song to initiate courtship with females that are at a distance [54], the lack of physical proximity and a lower pulse song amplitude combine to seriously handicap the mutant male’s chances of copulation. These results demonstrate that DMLC2 plays an essential role in courtship and mating success and that sexual selection may place constraints on the evolution of this protein.

While questions remain about which specific pulse song component is most influential in female choice, several studies have indicated that PDC is more important than IPF and IPI [20], [22]. In the case of Dual males, a lower PDC (Figure 3F) is likely to further reduce mating competitiveness. Despite the reduction in PDC, Dual males outcompete Ext males who produce songs with normal PDC (Figure 3F) but longer pulses with more cycles (Figure 3B, C) that, given the female’s preference for robust pulse songs, should had conferred an advantage to Ext. However, this advantage is neglected by Ext males’ reluctance to engage in courtship as evidenced by their greatly suppressed CI and WEI, whether alone with a female (Figure 4) or in the presence of a competing male (Figure 5). Additionally, the sine song produced by Ext males has a much higher frequency than the sine song produced by any of the other three strains studied (Figure 2). While the role of the sine song is debatable [22], several studies have suggested that it serves to prime the female to copulation [52], [53], [55]. Regardless of which song or behavioral parameter is most important for successful courtship and copulation, our results are consistent in showing that the N-terminal extension may be under greater sexual selection constraints than the highly conserved phosphorylation sites (serine 66 and serine 67).

### Conclusion

Here, we show for the first time that mutations in a gene encoding a muscle contractile protein (myosin regulatory light chain) have distinct effects on courtship song and mating behavior. This adds a new category of genes known to influence *Drosophila* courtship that have specific effects in other physiological processes [56]. An N-terminal deletion of DMLC2 affects the sine and pulse elements of the song and impairs behaviors that are typical of the mating ritual. Mutations that abolish two phosphorylation sites also impair mating behavior but do not affect pulse song elements known to be important in conspecific mate choice [22]. Surprisingly, the defective song elements manifested in the single mutant lines are suppresed in Dual, a trangenic line expressing the N-terminal deletion and the phosphorylation sites mutations. This interaction is the opposite of that observed in flight mechanics and IFM structure in which the combination of the two types of mutation has an additive effect [24], [30]. These results suggest that the DMLC2 requirements for flight and song are different. This could be due to: (i) different functions of DMLC2 in flight and song; (ii) different requirements of the IFM in flight and song, either in magnitude or mechanism. In conclusion, mutations in *Dmlc2* (and perhaps other muscle protein genes) are pleiotropic with respect to flight and song. As these two behaviors are subject to different selection pressures, a comparative analysis of mutation effects on flight and song can reveal how individual genes respond to competing pressures and evolve under different selection mechanisms.

## Materials and Methods

### *Drosophila* Lines Used

The wild type *D. melanogaster* stock is a laboratory strain of Oregon R (OR). The generation of the following transgenic strains used in this study has been previously described: (i) Control, strain expressing full length myosin regulatory light chain (D*mlc2* ^+^) [51]; Ext, strain expressing myosin regulatory light chain with truncated N-terminal extension (D*mlc2* ^Δ2–46^) [27]; Phos, strain expressing myosin regulatory light chain with disrupted myosin light chain kinase phosphorylation sites (D*mlc2* ^S66A,S67A^) [26]; Dual, strain expressing myosin regulatory light chain with both the phosphorylation and the N-terminal truncation mutations (D*mlc2* ^Δ2–46; S66A,S67A^) [30]. All transgenes are expressed in a myosin regulatory light chain null background. The mutant proteins that are expressed in each of the above-mentioned lines are pictorially diagrammed in Figure 1 in [24]. The flies were raised in standard cornmeal food and maintained at 22°C and 70% humidity in a room with 12∶12 hr light:dark cycles.

(see http://stockcenter.ucsd.edu/info/food_cornmeal.php for ingredients and recipe).

### Flight Performance

Flight tests and wing-beat frequency analysis were performed as previously described [57].

### Courtship Song Recording

Virgin males and females were collected using CO_2_. Males were aspirated into single vials and kept isolated for 24 hrs before testing so as to nullify any grouping effect and to increase amount of song production [58]. Males aged 3 days and females aged 24 hrs or less were used for courtship song assays to stimulate the males to produce more songs. A male and a female were aspirated into a small plexiglass mating chamber (1 cm diameter × 4 mm height) and placed inside an INSECTAVOX [59] for song recording for 30 minutes duration. For details, see [60].

### Courtship Song Analysis

The recorded songs from the INSECTAVOX were extracted either with Audacity (http://audacity.sourceforge.net/) or directly digitized using Goldwave v5.58 software [61]. The digitized waveform of the recorded songs were then logged and manually analyzed in Goldwave v5.58 to extract courtship song parameters. For list of song parameters and details of courtship song analysis procedure, see Table S1 and [60].

### Single Pair Mating Assay

Three to five day old virgin males and females were used. Each assay consisted of one male of a transgenic strain and one wild type (Oregon R) female introduced into a plexiglass mating chamber (1 cm diameter × 4 mm height). The courtship activities were video recorded until successful copulation, or longer (30–50 minutes) in the absence of copulation, using a 65X SD camcorder (Samsung) mounted on a tripod. The assays were done under room light at 22°C temperature and 70% humidity. From the videos, CI and WEI were calculated for each male as described in [13]. Briefly, CI is the fraction of the total recording time the male displayed courtship behaviors (orienting, chasing, tapping, licking, singing, copulation attempts), and WEI is the fraction of the total recording time the male extends and vibrates a wing for singing (Table S1).

### Courtship Competition Assay

Three to five day old virgin males and females were used. Twenty four hours before testing, the males were anesthetized with CO_2_ and one of them was marked on its thorax with a neon-orange paint using a fine point paintbrush. Two males (one marked and one unmarked) of different transgenic strains and one wild type OR female were introduced into a plexiglass mating chamber (1 cm diameter× 4 mm height) and courtship activities were video recorded for 30–50 minutes using a 65X SD camcorder (Samsung) mounted on a tripod (Vanguard). The assays were done under light at 22°C temperature and 70% humidity. The competition videos were observed and the strain of the male that succeeded in courting and copulating with the wild type female was noted. Female preference index (FPI) was calculated as the relative advantage of the mutant male over the Control male (i.e., the excess copulations with the mutant male divided by the total number of copulations, [62]). When competing Ext to the other two mutants, FPI was calculated as the relative advantage of the non-Ext mutant males compared to Ext. Courtship index (CI) and wing extension index (WEI) were also calculated for each male as described in [13] to note the strain of the male that outcompeted the other in performing the courtship rituals.

### Statistical Analysis

All values are mean ± SE. Statistical analyses were performed using SPSS (v.20.0, SPSS, Chicago, IL), with values considered significant at p<0.05. One-way ANOVA followed by a post-hoc test by Fischer’s LSD pairwise comparisons between any two groups was used to examine differences between the Ext, Phos, Dual and Control for all variables. For statistical analysis on courtship song data, the average value of each song parameter was calculated for each fly; hence the number of statistical samples is the number of flies.

## Supporting Information

Table S1 **Male courtship song and behavioral parameters.**
(DOCX)

Audio S1 **Male courtship song (sine and pulse) sample of Control male in the presence of a wild type (Oregon R) female.**
(MP3)

Audio S2 **Male courtship song (sine and pulse) sample of Ext male in the presence of a wild type (Oregon R) female.**
(MP3)

Audio S3 **Male courtship song (sine and pulse) sample of Phos male in the presence of a wild type (Oregon R) female.**
(MP3)

Audio S4 **Male courtship song (sine and pulse) sample of Dual male in the presence of a wild type (Oregon R) female.**
(MP3)

Video S1 **Success of Control male over Ext male for wild type (Oregon R) female in courtship competition.**
(MP4)

Video S2 **Success of Control male over Phos male for wild type (Oregon R) female in courtship competition.**
(MP4)

Video S3 **Success of Control male over Dual male for wild type (Oregon R) female in courtship competition.**
(MP4)

Video S4 **Success of Phos male over Ext male for wild type (Oregon R) female in courtship competition.**
(MP4)

Video S5 **Success of Dual male over Ext male for wild type (Oregon R) female in courtship competition.**
(MP4)

## References

[1] Brodsky AK (1994) The evolution of insect flight. Oxford Univ Press: 248.

[2] Dudley R (2000) The evolutionary physiology of animal flight: paleobiological and present perspectives. Annu Rev Physiol 62: 135–155. 10845087 10.1146/annurev.physiol.62.1.135

[3] Greenfield MHD (1994) Cooperation and conflict in the evolution of signal interactions. Annu Rev Ecol Syst 25: 97–126.

[4] Ryna MJ, Rand AS (1993) Species recognition and sexual selection as a unitary problem in animal communication. Evolution 47: 647–657. 28568715 10.1111/j.1558-5646.1993.tb02118.x

[5] Gerhardt HC (1994) The evolution of vocalization in frogs and toads. Annu Rev Ecol Syst 25: 293–324.

[6] Hedwig B (2006) Pulses, patterns and paths: neurobiology of acoustic behaviour in crickets. J Comp Physiol A Neuroethol Sens Neural Behav Physiol 192: 677–689. 16523340 10.1007/s00359-006-0115-8

[7] Searcy WA, Andersson M (1986) Sexual selection and the evolution of song. Annu Rev Ecol Syst 17: 507–533.

[8] Ritchie MG, Phillips SDF (1998) The genetics of sexual isolation. In: Mindless Forms: Species and Speciation. Oxford University Press In: Howard, D A, Berlocher, S, editors: 291–308.

[9] Bennet-Clark HC (1971) Acoustics of insect song. Nature 234: 255–259.

[10] Spieth HT (1952) Mating behaviour within the genus Drosophila (Diptera). Bull Am Mus Nat Hist 99: 401–474.

[11] Ewing AW, Bennet-Clark HC (1968) The courtship songs of Drosophila. Behaviour 31: 288–301.

[12] Hall JC (1994) The mating of a fly. Science 264: 1702–1714. 8209251 10.1126/science.8209251

[13] Ejima A, Griffith LC (2007) Measurement of courtship behavior in Drosophila melanogaster. Cold Spring Harb Protoc. 10.1101/pdb.prot4847 21356948

[14] Markow TA, O’Grady PM (2005) Evolutionary genetics of reproductive behavior in Drosophila. Annual Review of Genetics 39: 263–291. 10.1146/annurev.genet.39.073003.112454 16285861

[15] Ewing AW, Bennet-Clark HC (1977) The Neuromuscular Basis of Courtship Song in Drosophila: The Role of the Indirect Flight Muscles. J Comp Physiol 119: 249–265.

[16] Ewing AW (1979) Neuromuscular basis of courtship song in Drosophila: the role of the direct and axillary wing muscles. Journal of Comparative Physiology 130: 87–93.

[17] Josephson RK, Malamud JG, Stokes DR (2000) Asynchronous muscle: a primer. J Exp Biol 203: 2713–2722. 10952872 10.1242/jeb.203.18.2713

[18] Bennet-Clark HC, Ewing AW (1968) The wing mechanism involved in the courtship of Drosophila. J Exp Biol 49: 117–128.

[19] Wheeler DA, Kulkarni SJ, Gailey DA, Hall JC (1989) Spectral analysis of courtship songs in behavioral mutants of Drosophila melanogaster. Behav Genet 19: 503–528. 2508613 10.1007/BF01066251

[20] Rybak F, Aubin T, Moulin B, Jallon JM (2002) Acoustic communication in Drosophila melanogaster courtship: are pulse- and sine-song frequencies important for courtship success? Can J of Zool 80: 987–996.

[21] Wheeler DA, Fields WL, Hall JC (1988) Spectral analysis of Drosophila courtship songs: D. melanogaster, D. simulans, and their interspecific hybrid. Behavior Genetics 18: 675–703. 3146969 10.1007/BF01066850

[22] Talyn BC, Dowse HB (2004) The role of courtship song in sexual selection and species recognition by female Drosophila melanogaster. Anim Behav 68: 1165–1180.

[23] Lehmann FO, Dickinson MH (1997) The changes in power requirements and muscle efficiency during elevated force production in the fruit fly Drosophila melanogaster. J Exp Biol 200: 1133–1143. 9131808 10.1242/jeb.200.7.1133

[24] Miller MS, Farman GP, Braddock JM, Soto-Adames FN, Irving TC, Vigoreaux JO, Maughan DW (2011) Regulatory light chain phosphorylation and N-terminal extension increase cross-bridge binding and power output in Drosophila at in vivo myofilament lattice spacing. Biophys J 100: 1737–1746. 21463587 10.1016/j.bpj.2011.02.028 PMC3072621

[25] Parker VP, Falkenthal S, Davidson N (1985) Characterization of the myosin light- chain-2 gene of Drosophila melanogaster. Mol Cell Biol 5: 3058–3068. 3018498 10.1128/mcb.5.11.3058-3068.1985 PMC369119

[26] Tohtong R, Yamashita H, Grahan M, Haeberle J, Simcox A, Maughan D (1995) Impairment of muscle functions caused by mutations of phosphorylation sites in myosin regulatory light chain. Nature 374: 650–653. 7715706 10.1038/374650a0

[27] Moore JR, Dickinson MH, Vigoreaux JO, Maughan DW (2000) The effect of removing the N-terminal extension of the Drosophila myosin regulatory light chain upon flight ability and the contractile dynamics of indirect flight muscle. Biophys J 78: 1431–1440. 10692328 10.1016/S0006-3495(00)76696-3 PMC1300741

[28] Dickinson MH, Hyatt CJ, Lehmann F, Moore JR, Reedy MC, Simcox A, Tohtong R, Vigoreaux JO, Yamashita H, Maughan DW (1997) Phosphorylation dependent power output of transgenic flies: an integrated study. Biophys J 73: 3122–3134. 9414224 10.1016/S0006-3495(97)78338-3 PMC1181215

[29] Irving T, Bhattacharya S, Tesic I, Moore J, Farman G, Simcox A, Vigoreaux J, Maughan D (2001) Changes in myofibrillar structure and function produced by N-terminal deletion of the regulatory light chain in Drosophila. J Muscle Res Cell Motil 22: 675–683. 12222828 10.1023/a:1016336024366

[30] Farman GP, Miller MS, Reedy MC, Soto-Adames FN, Vigoreaux JO, Maughan DW, Irving TC (2009) Phosphorylation and the N-terminal extension of the regulatory light chain help orient and align the myosin heads in Drosophila flight muscle. J Struct Biol 168: 240–249. 19635572 10.1016/j.jsb.2009.07.020 PMC2757514

[31] Tauber E, Eberl DF (2001) Song production in auditory mutants of Drosophila: the role of sensory feedback. J Comp Physiol A 187: 341–348. 11529478 10.1007/s003590100206

[32] Bennet-Clark HC, Dow M, Ewing AW, Manning A, von Schilcher F (1976) Letter: Courtship stimuli in Drosophila melanogaster. Behav Genet 6: 93–95. 814891 10.1007/BF01065681

[33] Swank DM, Vishnudas VK, Maughan DW (2006) An exceptionally fast actomyosin reaction powers insect flight muscle. Proc Natl Acad Sci U S A 103: 17543–17547. 17085600 10.1073/pnas.0604972103 PMC1859965

[34] Iwamoto H, Nishikawa Y, Wakayama J, Fujisawa T (2002) Direct x-ray observation of a single hexagonal myofilament lattice in native myofibrils of striated muscle. Biophys J 83: 1074–1081. 12124287 10.1016/S0006-3495(02)75231-4 PMC1302209

[35] Iwamoto H, Inoue K, Yagi N (2006) Evolution of long-range myofibrillar crystallinity in insect flight muscle as examined by X-ray cryomicrodiffraction. Proc Biol Sci 273: 677–685. 16608686 10.1098/rspb.2005.3389 PMC1560076

[36] Gordon S, Dickinson MH (2006) Role of calcium in the regulation of mechanical power in insect flight. PNAS 103: 4311–4315. 16537527 10.1073/pnas.0510109103 PMC1449689

[37] Gotz KG, Hengstenberg B, Biesinger R (1979) Optomotor control of wing beat and body posture in Drosophila. Biol Cybernetics 35: 101–112.

[38] Gotz KG, Wandel U (1984) Optomotor control of the force of flight in Drosophila and Musca. II. Covariance of lift and thrust in still air. Biol Cybernetics 51: 135–139.

[39] Dickinson MH, Lighton JRB (1995) Muscle efficiency and elastic storage in the flight motor of Drosophila. Science 128: 87–89. 10.1126/science.7701346 7701346

[40] Nachtigall W, Wilson DM (1967) Neuromuscular control of dipteran flight. J Exp Biol 47: 77–97. 6058982 10.1242/jeb.47.1.77

[41] Wang Q, Zhao C, Swank DM (2011) Calcium and stretch activation modulate power generation in Drosophila flight muscle. Biophys J 101: 2207–2213. 22067160 10.1016/j.bpj.2011.09.034 PMC3207158

[42] Lehmann F-O, Dimitri S, Berthe R (2013) Calcium signaling indicates bilateral power balancing in the Drosophila flight muscle during maneuvering flight. J R Soc Interface 10: 20121050. 23486171 10.1098/rsif.2012.1050 PMC3627081

[43] Krzic U, Rybin V, Leonard KR, Linke WA, Bullard B (2010) Regulation of oscillatory contraction in insect flight muscle by troponin. J Mol Biol 397: 110–118. 20100491 10.1016/j.jmb.2010.01.039

[44] Qiu F, Lakey A, Agianian B, Hutchings A, Butcher GW, Labeit S, Leonard K, Bullard B (2003) Troponin C in different insect muscle types: identification of two isoforms in Lethocerus, Drosophila and Anopheles that are specific to asynchronous flight muscle in the adult insect. Biochem J 371: 811–821. 12558500 10.1042/BJ20021814 PMC1223341

[45] Campbell K (2006) Filament compliance effects can explain tension overshoots during force development. Biophys J 91: 4102–4109. 16950846 10.1529/biophysj.106.087312 PMC1635681

[46] Luo Y, Cooke R, Pate E (1993) A model of stress relaxation in cross-bridge systems: effect of a series elastic element. Am J Physiol 265: C279–C288. 8338135 10.1152/ajpcell.1993.265.1.C279

[47] Tanner BC, Daniel TL, Regnier M (2012) Filament compliance influences cooperative activation of thin filaments and the dynamics of force production in skeletal muscle. PLoS Comput Biol 8: e1002506. 22589710 10.1371/journal.pcbi.1002506 PMC3349719

[48] Tanner BCW, Daniel TL, Regnier M (2007) Sarcomere lattice geometry influences cooperative myosin binding in muscle. PLoS Comput Biol 3: e115. 17630823 10.1371/journal.pcbi.0030115 PMC1914368

[49] Lowey S, Waller GS, Trybus KM (1993) Function of skeletal muscle myosin heavy and light chain isoforms by an in vitro motility assay. J Biol Chem 268: 20414–20418. 8376398

[50] Sweeney HL (1995) Function of the N terminus of the myosin essential light chain. Biophys J 68: 112s–119s. 7787052 PMC1281889

[51] Warmke J, Yamakawa M, Molloy J, Falkenthal S, Maughan D (1992) Myosin light chain-2 mutation affects flight, wing beat frequency, and indirect flight muscle contraction kinetics in Drosophila. J Cell Biol 119: 1523–1539. 1469046 10.1083/jcb.119.6.1523 PMC2289745

[52] Kowalski S, Aubin T, Martin J-R (2004) Courtship song in Drosophila melanogaster: a differential effect on male–female locomotor activity. Can J Zool 82: 1258–1266.

[53] Schilcher Fv (1976) The function of pulse song and sine song in the courtship of Drosophila melanogaster. Anim Behav 24: 622–625.

[54] Trott AR, Doneslson NC, Griffith LC, Ejima A (2012) Song choice is modulated by female movement in Drosophila males. PLoS ONE 7. 10.1371/journal.pone.0046025 PMC3458092 23049926

[55] Crossley SA, Bennet-Clark HC, Evert HT (1995) Courtship song components affect male and female Drosophila differently. Animal Behaviour 50: 827–839.

[56] Mackay TF, Heinsohn SL, Lyman RF, Moehring AJ, Morgan TJ, Rollmann SM (2005) Genetics and genomics of Drosophila mating behavior. Proc Natl Acad Sci U S A 102 Suppl 16622–6629. 15851659 10.1073/pnas.0501986102 PMC1131870

[57] Vigoreaux JO, Hernandez C, Moore J, Ayer G, Maughan D (1998) A genetic deficiency that spans the flightin gene of Drosophila melanogaster affects the ultrastructure and function of the flight muscles. J Exp Biol 201: 2033–2044. 9622575 10.1242/jeb.201.13.2033

[58] Noor MAF, Aquadro CF (1998) Courtship songs of Drosophila pseudoobscura and D. persimilis: analysis of variation. Anim Behav 56: 115–125. 9710468 10.1006/anbe.1998.0779

[59] Gorczyca M, Hall JC (1987) The INSECTAVOX, an integrated device for recording and amplifying courtship songs. Drosophila Information Service 66: 157–160.

[60] Chakravorty S, Wajda MP, Vigoreaux JO (2012) Courtship song analysis of Drosophila muscle mutants. Methods 56: 87–94. 21945578 10.1016/j.ymeth.2011.09.007

[61] Goldwave Inc.(2010) St John’s, Newfoundland, Canada.

[62] Demir E, Dickson BJ (2005) Fruitless splicing specifies male courtship behavior in Drosophila. Cell 121: 785–794. 15935764 10.1016/j.cell.2005.04.027

