# Supplementary material for: Mutations of the Drosophila Myosin Regulatory Light Chain Affect Courtship Song and Reduce Reproductive Success
Source: PLoS One. 2014 Feb 26;9(2):e90077. doi: 10.1371/journal.pone.0090077 (PMC3935995; doi:10.1371/journal.pone.0090077)
Supplement: Table S1 — Male courtship song and behavioral parameters. (DOCX) [file pone.0090077.s001.docx]

**Table 1. Male courtship song and behavioral parameters**

| **Parameter** | **Abbreviation** | **Description** |
| --- | --- | --- |
| Sine song duration | SDUR | Time duration (ms) of a sine song burst. |
| Sine song frequency | SSF | Carrier frequency (Hz) of a sine song burst. |
| Pulse duty cycle | PDC | Equivalent to the ratio of pulse song to the total time of recording (song + silence). |
| Pulse length | PL | Number of pulses in a pulse song train. |
| Cycles per pulse | CCP | Number of zero crossings by the pulse waveform divided by two. |
| Intrapulse frequency | IPF | Carrier frequency (Hz) of a pulse. |
| Interpulse interval | IPI | Time duration (ms) between the equivalent peaks of two consecutive pulses in a train. |
| Courtship index | CI | Fraction of the total recording time the male displayed courtship behaviors (orienting, chasing, tapping, licking, singing, copulation attempts). |
| Wing extension index | WEI | Fraction of the total recording time the male extends and vibrates a wing for singing. |
| Female preference index | FPI | The relative advantage of one male (e.g., mutant) over another male (e.g., control); the excess copulations with the mutant male divided by the total number of copulations. |
